# Editorial: Coronary Artery Anomalies: A 2020 Review

**DOI:** 10.3389/fcvm.2022.776951

**Published:** 2022-02-10

**Authors:** Christoph Gräni, Massimo Antonio Padalino

**Affiliations:** ^1^Department of Cardiology, Inselspital, Bern University Hospital, University of Bern, Bern, Switzerland; ^2^Department of Cardiac, Thoracic and Vascular Sciences, and Public Health, University of Padova, Padua, Italy

**Keywords:** ACAOS, anomalous aortic origin of the coronary artery (AAOCA), coronary artery anomalies (CAA), anomalous coronary arteries originating from the opposite sinus of Valsalva, anomalous coronary arteries

Coronary artery anomalies (CAA) are one of the remaining mysteries in cardiology. Currently, reports of an anomalous coronary vessel origin/and or course is rare (1), but with the increasing accuracy of the latest imaging technology, they may begin to be found more often. However, their clinical significance varies greatly across different variants (2, 3). The spectrum ranges from the hemodynamically relevant anomalous origin of the left coronary artery from the pulmonary artery (3) to benign coincidental findings of a superficial myocardial bridge. Within that range, the anomalous coronary artery from the opposite sinus of Valsalva (ACAOS) with an interarterial course (i.e., course of the anomalous coronary tract between the aorta and the pulmonary artery) often leaves treating physicians in the dark, as the anatomical presence of ACAOS does not automatically imply a risk for future events. In the 90's and 2000's, important data from autopsy studies were published, showing that CAA may represent the underlying cause of sports-related sudden cardiac death in certain circumstances (4, 5). However, as this does not reflect the risk of adverse cardiac events in a person living (mostly unknowingly) with CAA, more data are needed to accurately counsel and treat these patients in this clinical setting. Moreover, as invasive and noninvasive imaging becomes more relevant with consecutive increasing cases of CAA, evidence on optimal downstream testing and therapy is needed in order to tailor therapy. Lastly, despite the reported low surgical risk for most repair procedures for CAA, a real benefit in minimizing the sudden death risk has not been fully demonstrated yet. In this regard, this special section “*Coronary Artery Anomalies: A 2020 Review”* in Frontiers in Cardiovascular Medicine aims to address some of the major questions in the setting of CAA. We thank the authors for their eminent contributions and it is with great pleasure that we can present eight interesting articles on this important topic from different internationally recognized research groups in the field of CAA.

We congratulate the authors Thiene et al. and Rizzo et al., both from the University of Padua, for the two first articles. They nicely summarize the history, the embryology, and anatomical aspects of CAA by illustrating different aspects with historical pictures, excellent drawings, images from postmortem casts, and pathological specimens. The anatomy of subepicardial coronary arteries in normal hearts and its right, left, or co-dominance was first published in a milestone paper in 1903 by Antonio Banchi, an anatomist in Florence, and then confirmed by post-mortem casts in 1963 by Giorgio Baroldi and shortly after *in vivo* by Sones at the Cleveland clinic. From the embryology point of view, the supepicardial vessels derive from extracardiac epicardial cells, which form a plexus like vasculature secondary invading the myocardium and growing toward the aortic root to the facing aortic sinuses (Thiene et al.). During this process a misconnection to the wrong sinus of the Valsalva can occur, however, its exact pathological mechanisms seem to be still not well-understood. Four rare anomalies are nicely presented and discussed in different clinical settings in the articles published by Borns et al. and Tang et al.. One case was a 7-year-old girl diagnosed with a single left coronary ostium with a giant coronary trunk, coronary artery to right ventricle fistula, and coronary artery ring. The authors documented the case very well and it is the first described variant of its kind in the literature. The coronary fistula was surgically ligated with an off-pump strategy and the patient was discharged on day five post-operation and free of symptoms during the 3 years of follow-up (Tang et al.). The three other cases by Borns et al. were a 14 years old teenager with left ACAOS with acute chest pain and elevated troponin, a 15-year old girl with a right ACAOS with chest pain, and an 11 year- old girl with syncope after swimming and ventricular fibrillation and an anomalous origin of the left coronary artery arising from the non-facing sinus without an interarterial course, but with a short intramural course. Low-dose computed tomography with 0.38 mSv and 60 ml contrast or cardiac magnetic resonance imaging were used in these cases and seem to be the optimal anatomical imaging modality to exactly describe the anatomy. As all these cases presented with acute signs and symptoms of ischemia due to the underlying CAA, timely surgical correction was performed. The three cases showed an intramural course and therefore the surgical approach was unroofing (Borns et al.). Interestingly enough, in the case with an anomalous coronary artery originating from the non-facing (non-coronary) sinus without an interarterial course, a long (i.e., 1 cm) intramural course was confirmed intraoperatively and therefore it seems that the anatomical high-risk feature of intramural course has to be considered separately from the interarterial course and is not always linked to it. In this regard, Bigler et al. illuminate in a comprehensive and in-depth review the pathophysiology of different anatomical high-risk features of CAA and how hemodynamic relevance should be assessed. The concept of a fixed component, similar to the stenosis known from coronary artery disease (anatomic high-risk features of slit-like ostium and proximal narrowing) and additional dynamic component (acute take-off angle, intramural course with the elliptic vessel shape) is discussed. It is highlighted that patients with a CAA should not be invariably referred to direct surgery, but rather should undergo a thorough noninvasive and invasive assessment. This includes an anatomic description of the anatomy (best done with computed tomography or cardiac magnetic resonance) and additional functional testing with dobutamine/volume challenge under maximal heart rate (i.e., invasive fractional flow reserve evaluation and intravascular ultrasound imaging to depict the presence and extent of possible dynamic lateral compression) (Bigler et al.). However, the authors emphasize that besides the assessment of ischemia, one has to be aware of the possible presence of myocardial fibrosis and scarring (e.g., suspected to occur in anomalies as an expression of recurrent myocardial ischemia) which may serve as the substrate for ventricular tachyarrhythmia's, and has to be taken into account for risk stratification of patients with CAA. Regarding the optimal surgical approach, Padalino et al. elaborate a complete review on the various surgical techniques (coronary unroofing, osteoplasty, reimplantation, pulmonary artery translocation, coronary artery bypass grafting), which are usually devoted to a particular anatomical subtype of CAA. Although ideally, the coronary surgical reconstruction for CAA should normalize the anatomy, relocating the large ostium in the center of the appropriate sinus, reproducing a normal take-off angle, and eliminating any intramural or interarterial course, none of the current surgical techniques can address all of these components, and each is susceptible to individual technical pitfalls (Padalino et al.). To document and control treatment success, Meijer et al., propose a compelling imaging approach in their original study. In detail, 11 patients were retrospectively identified out of 54 consecutive patients who underwent surgical repair of CAA over a 17 year period at the Leiden University Medical Center, and who had pre- and post-operative computed tomography imaging available. The origin and course of the anomalous coronary artery and the ostial dimensions were evaluated and correlated with restenosis of the operated coronary artery. To allow an accurate evaluation of the effective orifice area at diagnosis and after surgical repair introduced a new parameter—the coronary triangulated orifice area (CTOA). In fact, postoperatively, the median CTOA increased significantly from 1.6 mm^2^ [IQR 0.9;4.9] to 5.5 mm^2^ [IQR 3;11.8] (*p* < 0.005). During follow-up, in three patients restenosis of the operated coronary artery was suspected and in these patients the CTOA showed only a limited postoperative increase of ≤ 1.4 mm^2^. This study highlights, that patients with CAA undergoing surgery should be closely monitored and possibly benefit from post-operative imaging to derive the new anatomic circumstances. Besides surgery, percutaneous coronary intervention (PCI) might be another alternative approach in selected patients with CAA. In an interesting overview by Aubry et al. the role of PCI in patients with CAA and interarterial course is presented and discussed. There is not much literature published on PCI in CAA so far, and we congratulate the authors for establishing the ANOCOR working group which, since 2014, has started a prospective registry of patients with right coronary artery anomalies and evaluated the outcome of those treated by PCI with stenting. The strengths of PCI are a shorter hospital stay and fewer post-procedural adverse events compared to surgery, however, long-term potency is not well-known and this approach might be rather a possible future strategy for older patients and not for younger adults or children. Furthermore, PCI may be a bail-out strategy to treat some surgery failures, such as acute occlusion or scarring stenosis (Aubry et al.).

To conclude, within the last decades we have gained important knowledge and evidence on coronary artery anomalies. However, large-scale prospective studies are still lacking, and hopefully, the efforts of in-depth systematic assessment of CAA like the NARCO (ClinicalTrials.gov ID: NCT04475289) and MuSCAT trials (6), and several multinational registries (7, 8) will provide a better understanding of CAA and its risks. Hopefully, the new evidence can help to guide physicians in the future toward the optimal selection of surgical candidates and adequate surgical techniques. This would reduce the incidence of adverse cardiac events, but also could help avoid unnecessary open-heart surgery in low-risk patients.

## Author Contributions

All authors listed have made a substantial, direct, and intellectual contribution to the work and approved it for publication.

## Funding

This work was supported by Swiss National Science Foundation (Grant Number 200871 to CG).

## Conflict of Interest

The authors declare that the research was conducted in the absence of any commercial or financial relationships that could be construed as a potential conflict of interest.

## Publisher's Note

All claims expressed in this article are solely those of the authors and do not necessarily represent those of their affiliated organizations, or those of the publisher, the editors and the reviewers. Any product that may be evaluated in this article, or claim that may be made by its manufacturer, is not guaranteed or endorsed by the publisher.
